# Children Exposed to Intimate Partner Violence During Confinement: Characteristics by Age and Sex

**DOI:** 10.3389/fpsyg.2022.889697

**Published:** 2022-06-20

**Authors:** Mavi Alcántara-López, Maravillas Castro, Antonia Martínez-Pérez, Visitación Fernández, Kaveri Negrón-Medina, Concepción López-Soler

**Affiliations:** ^1^Faculty of Psychology, University of Murcia, Murcia, Spain; ^2^Spanish Association for the Development of Mental Health in Childhood and Youth, “I Want to Grow”, Murcia, Spain

**Keywords:** COVID-19, confinement, children, age, sex, intimate partner violence (IPV)

## Abstract

The COVID-19 pandemic and restrictions imposed to stop its advance have affected the entire population. Children living with difficulties or in vulnerable situations prior to the pandemic might have suffered an even greater impact. This present study examines the psychological impact of quarantine on children and adolescents exposed to intimate partner violence against their mothers. Participants were 185 mothers who reported 269 children, as well as 108 children who self-reported. An emotional and behavioral checklist was administered to both mothers and children throughout confinement. Results show mothers observed changes in their children’s psychological state. Children, in turn, reported an increase in different variables. Mothers reported a higher percentage of overall increase for both general and severe symptoms than their children. Differences were found by sex and age. Future research with similar population groups is necessary to establish the support and intervention children require in similar contexts, as well as to clarify possible causes of differences found by age and sex.

## Introduction

The impact of confinement on mental health is being studied in order to discover its psychological effects on different population groups (Martínez de Salazar and López-Soler, 2020; Primdahl et al., 2021). In the adult population, it has been found that anxiety, depression, and particularly stress levels all increased significantly during confinement (Planchuelo-Gómez et al., 2020; Ayuso-Mateos et al., 2021; González-Sanguino et al., 2021; Gilbar et al., 2022; Medda et al., 2022; Niederkrotenthaler et al., 2022). There are direct and indirect effects derived from confinement that can affect the mental health of the child and adolescent population: thus, the former would be linked to psychological detriment caused by the reduction in social interaction and the latter with the negative impact on the mental health of family members and primary caregivers (Robertson et al., 2021; Sonuga-Barke and Fearon, 2021).

### Effects of COVID-19 on the Community Child Population

Various studies conducted worldwide have confirmed the impact of COVID-19 on the mental health of children and adolescents, according to information provided by parents.

Jiao et al. (2020) found an increase in irritability and fear among Chinese children. Weissman et al. (2021) analyzed a longitudinal sample (*N* = 145, age range, 10–15 years) at 3 crucial moments: before COVID-19, at the start of confinement, and 6 months later. Results showed that greater exposure to pandemic-related stressors was linked to greater internalizing problems both at the beginning and in the following assessments. Pisano et al. (2020) performed a study with Italian children (4–10 years old), and reported 26.48% presented regressive symptoms, 18.17% showed more fears, 53.53% were more irritable, 21.17% exhibited mood swings, and 19.99% more sleep problems. Bignardi et al. (2021) in measures offered by parents found an increase in depressive but not anxious symptomatology in ages between 7 and 11. Wright et al. (2021), assessed the impact of COVID-19 on the mental health of children and their mothers. They employed measurements taken both during 3 months prior to the COVID-19 pandemic and during 3 months following imposition of lockdown, finding that, in the United Kingdom, confinement was associated with a significant increase in adolescent mental health problems. According to measures provided by both children and their mothers, depression levels in children under 11 and 12 years of age increased during confinement between 44 and 71%, respectively. Mothers reported a 44% increase in disruptive behavior problems, but not in anxiety levels. The German COPSY study (impact of COVID-19 on psychological health) by Ravens-Sieberer et al. (2021), took measurements during partial lockdown in Germany (May 26 to June 10, 2020). Using information provided by parents, an increase was found in mental health problems in sons and daughters aged between 7 and 17 years. Therefore, the prevalence of mental health problems was 9.9% (*n* = 153) prior to the pandemic and increased to 17.8% (*n* = 283) during. Among problems reported by parents we found hyperactivity during the pandemic [*n* = 233 (14.6%)], emotional problems [*n* = 210 (13.3%)], peer problems [*n* = 183 (11.5%)], and behavioral problems [*n* = 159 (10.0%)].

Likewise, different reviews revealed a high incidence of emotional problems in this population (Deolmi and Pisani, 2020; Imran et al., 2020; Nearchou et al., 2020; Ma et al., 2021).

Also in Spain, in a recent review by Amorós-Reche et al. (2022), it was concluded that mental health problems increased overall during confinement; Berasategi et al. (2021) compiled information, after 3–4 weeks of confinement, from 1,046 Spanish parents with children between 2 and 14 years old, finding that 74.66% were more angry than usual, 55.54% cried more, 70.17% were more nervous, and 35.95% suffered more nightmares.

### Age and Sex Differences

Most works carried out to date conclude that age might be considered a risk factor for the development of different psychological disorders following COVID-19. Diverse results were found in research with samples of schoolchildren and adolescents. Studies generally report higher levels of anxiety, depression and emotional self-regulation problems in adolescents compared to schoolchildren (Lavigne-Cerván et al., 2021; Pizarro-Ruiz and Ordóñez-Camblor, 2021). While others, such as Pearcey et al. (2020), Gilsbach et al. (2021), and Ravens-Sieberer et al. (2021), consider that the increase in emotional and behavioral alterations is greater at ages below 11 years.

Some studies have reported sex differences; highlighting that girls generally have higher levels of anxiety, depression, and stress than boys (Duan et al., 2020; Tamarit et al., 2020; Zhou et al., 2020; Carrillo-López et al., 2021; Gilsbach et al., 2021), aspects confirmed by the systematic review by Panchal et al. (2021).

### Population With Psychosocial Vulnerability

In addition to the appearance of symptoms in children and adolescents in the community population, the situation caused by the pandemic can aggravate existing mental health problems in clinical contexts (Golberstein et al., 2020). In fact, some studies have highlighted the need to pay particular attention to those with previous mental health disorders, women and young people, as well as people living alone or in uncomfortable living conditions or at risk of social exclusion (Ali et al., 2021; González-Sanguino et al., 2021; López-Serrano et al., 2021; Pérez et al., 2021; Stevens et al., 2021). Gilsbach et al. (2021) in their comparative study of the psychological effects of the pandemic on children with or without mental health disorders found that the former exhibited a greater negative psychological impact than peers with good mental health. Girls and children with a depressive disorder were particularly at risk of psychological disorders associated with the pandemic.

Nevertheless, there is no sufficient body of empirical evidence on the impact of the pandemic on vulnerable children and adolescents, and the extent of the effects on their mental health is unknown, in addition to what risk factors are operating and which helped resilience during COVID-19 (Tso et al., 2022). Among the few published studies, Janiri et al. (2021) researched the relationship between psychological disorders and childhood trauma in 500 healthy participants during lockdown in Italy. Their main results showed that childhood trauma was associated with an increase in vulnerability to the stressful effect of COVID-19. They pointed out that emotional dysregulation might be the dimension that mediates the impact of trauma, aggravating negative post-confinement symptoms; particularly in children with a history of emotional abuse and/or neglect.

Within the massive effect of the pandemic on families, special attention has been paid to women and children at risk of or who have suffered gender-based violence, especially those who still live with the aggressor (Donagh, 2020; Usher et al., 2020). Some authors were concerned and argued that the pandemic could hinder filing a complaint, abandonment of the home or also make it difficult for women to seek help (Bradbury-Jones and Isham, 2020; Usher et al., 2020). However, during lockdown, reporting may have been less frequent as perpetrators were less likely to escape (Chatterjee et al., 2020).

Another impact level of this problem is related to the affectation of children exposed to intimate partner violence (IPV) in its various forms (Holden, 2003), whose consequences are widely documented, highlighting detrimental effects in all areas of development-emotional, social, cognitive, and academic (Loise, 2009; Alcántara et al., 2013; Maddoux et al., 2016; Castro et al., 2017; Rocha et al., 2021; Herrenkohl et al., 2022). Current studies focus on understanding and explaining what factors can mediate or moderate these consequences, as well as the different trajectories they follow throughout child and adolescent development (Bender et al., 2022; Evans et al., 2022; Lee et al., 2022).

In the context of this pandemic, it is important to verify the psychological effect of confinement on children with a prior history of exposure to gender-based violence. Evidence suggests that cumulative exposure to psychosocial adversity (experiences including childhood abuse and neglect, witnessing family violence, or other significant psychosocial stressors) can influence cognitive, socioemotional, and physical developmental processes (Lamers-Winkelman et al., 2012; Bethell et al., 2017). In the study by Guo et al. (2020), it was found that the largest variation in psychological disorders related to post-traumatic stress was explained by adverse experiences in childhood, with a higher number of experiences of abuse as a predictor of greater symptomatology of post-traumatic stress and other psychological disorders, concluding that following exposure to COVID-19 these can increase significantly.

In accordance with the study by Ravi et al. (2021) on the experiences of women survivors of gender-based violence in the context of COVID-19, the authors state that though their article is not focused on children, future research on this population is necessary (Ravi et al., 2021). Thus, this population group is our target in this study; given the scarcity of evidence on this risk population (Martínez-Pérez et al., 2020).

Likewise, research to date suggests that variables such as age and sex may be risk factors in being affected by IPV, as well as in the development of psychological disorders overall and following the impact of COVID in particular.

Therefore, the general aim of this study is to examine how the impact of COVID on the population exposed to IPV can increase previous psychological disorders. As a specific aim, it is analyzed whether there are sex and age differences in relation to symptomatology.

It is hoped that implications derived from this research contribute to early detection of worsening of prior symptoms, in order to prevent symptoms becoming chronic throughout their development. In addition to verifying whether sex and age variables provide more information that helps improve treatments, enabling an adaptation based on these characteristics.

## Materials and Methods

### Participants

Selection of mothers and children was carried out through incidental non-probability sampling. The children and/or their mothers were in a Psychological Care Service for Children of Women Victims of Gender Violence, previously referred by the Specialized Care Centers for Women Victims of Gender Violence. The study involved 185 mothers who reported on 269 children aged 0–18 years (M = 9.65; SD = 3.92) (Tables 1, 2). In addition, 108 of these children completed the emotional and behavioral checklist (*n* = 108), of whom 79.41% were 6 years old or above, 51.8% (*n* = 56) were boys, 48.2% (*n* = 52) girls, and 63.2% had visits with their father (most suspended during lockdown). Mothers’ ages ranged from 23 to 62 years (M = 40.5; SD = 7.01), 89.7% were Spanish. Of the 185 mothers, 1.6% had no education, 30.6% had completed primary, 48.1% secondary and 19.7% university education, and 41.8% were unemployed. At time of assessment for this study, 96.2% were not living with their aggressor, and of these, 21% had a partner. The women had varying amounts of children ranging between 1 and 10, 35.7% of mothers had requested psychological care for more than one child in the service, and in 36.75% assessment was made on more than one child. Likewise, in 59.2% of cases, children lived alone with the mother and 56.1% of the children had at least one sibling participating in the study. I order to verify the existence of affective and/or behavioral problems derived from confinement in different evolutionary stages (childhood and adolescence), the sample was reduced and disaggregated into two age groups: group 1 comprising 73 children between 6 and 12 years old (67.6%), and group 2 with 35 children between 13 and 18 years old (32.4%).

**TABLE 1:** Sociodemographic data of mothers.

		Mothers (185) M (SD)/%
Age	–	40.5 (7.01)
Nationality	Spanish	89.7
	Other	10.3
Education	No education	1.6
	Primary education	30.6
	Secondary education	48.1
	University education	19.7
Children participating in study	One	63.25
	More than one	36.75
Family Situation	With the aggressor	3.8
	Without the aggressor	96.2
Work	Yes	58.2
	No	41.8

**TABLE 2:** Sociodemographic data of children who self-report.

		Children (108) %
Age	<6	20.6
	>6	79.4
Sex	Boys	48.2
	Girls	51.8
Visits with father	Yes	63.2
	No	36.8

### Instruments

The instrument employed to assess the impact of confinement due to the COVID-19 health crisis was designed following review of the literature on the psychological impact of confinement and other traumatic events on children. For the current study, an instrument developed by the group was specifically used, generating an emotional and behavioral checklist for children during the COVID-19 state of alarm. The list comprised 35 symptoms assessing psychopathological indicators from 0 (not at all compared to before confinement) to 3 (a lot compared to before confinement). The internal consistency of the checklist was high for both mothers (α = 0.919) and children (α = 0.844).

### Procedure

The emotional and behavioral checklist was administered telephonically by a psychologist specialized in childhood psychology and adolescence.

The same instrument was used for mothers and children to evaluate the perception of both informants regarding changes that may have occurred regarding the children’s previous and current symptoms. Mothers were specifically asked about their perception of change in different symptoms in their child in the context of social restriction due to confinement. Children were asked if they had perceived any change in their previous symptoms since they had been obliged to stay home during lockdown, whether there had been variation in intensity of sadness or impulsiveness or whether they had experienced more worry.

The calls began 2 weeks after the state of alarm declared in Spain (March 14, 2020), and data was gathered over a period of 5 weeks (from March 31 to April 30, 2020). Informed consent was obtained from legal guardians of children who participated in the study, at all times guaranteeing its voluntary and confidential nature. An exclusion criterion was children who presented intellectual disability, autistic spectrum disorder, or psychotic disorder.

### Statistical Analysis

To achieve the study aim the research design was exploratory cross-sectional study with clinical sample.

For statistical treatment of data, the computerized statistical package IMB-SPSS-STATISTICS version 24 was used. Analyses carried out were as follows:

(a) Reliability analysis of the checklist used to evaluate clinical symptoms (Cronbach alpha).
(b) The exploratory analysis of basic data and basic descriptions of central tendency (maximum, minimum, and medium) and dispersion (standard deviation) was used to verify sample data and frequency distribution.
(c) Frequency analysis and contingency tables was carried out to find the prevalence of symptomatology in the two sample groups. The average difference was analyzed to relate the nominal and ordinal/continuous variables using parametric and non-parametric statistics (Pearson *Chi-square* contingency coefficient, V *Cramer* and *Fisher’s exact test*).
(d) Non-parametric tests were performed for independent samples, the *Mann-Whitney test* was used in variables with two levels, while the *Kruskal-Wallis H test* in variables with three or more levels. The *Wilcoxon* signed rank test was carried out for related samples and Spearman’s *Rho* correlation.

## Results

### Mothers’ Perception of the General Emotional and/or Behavioral Effects on Their Children During Confinement

Global prevalence was calculated from the sum of the scores on the Likert type scale (1 = somewhat, 2 = quite a lot, 3 = a lot) (Table 3). Mothers observed changes in the emotional and/or behavioral state of their children during confinement due to COVID-19, with an increase in 17 of the 35 indicators of symptoms: worry/nervousness (36.8%); sleep difficulties (24.9%); PTSD symptomatology and fear (24.5%); maladjustment and personal inadaptation (21.9%); nightmares (19.7%) and worry (19.3%); low tolerance to frustration (17.8%); emotional instability (16.7%); sadness and dependence on the mother (15.6%); Inadaptation in relationship with siblings (14.9%); dysphoria (13%); disruptive behaviors (12.6%) and aggressive (12.3%); attention problems (11.2%); learning difficulties (10.8%); and maladjustment in relationship with the father during this period (10.4%).

**TABLE 3:** Perception of psychological alterations in children during confinement by COVID-19.

Variables	Mothers’ perception (*N* = 269)						Children’ perception (*N* = 108)				
	f (%)	Boys *n* = 136	Girls *n* = 133	Age 0–5 *n* = 30	Age 6–12 *n* = 163	Age 13–18 *n* = 68	f (%)	Boys *n* = 56	Girls *n* = 52	Age 6–12 *n* = 73	Age 13–18 *n* = 35
		f (%)	f (%)	f (%)	f (%)	f (%)		f (%)	f (%)	f (%)	f (%)
Fear	35 (13)	24 (14.6)	11 (8.3)	4 (10.5)	28 (17.2)	3 (4.4)	13 (12)	9 (16.1)	4 (7.7)	8 (11)	5 (14.3)
Worry/nervousness	99 (36.8)	60 (44.1)	39 (29.3)	9 (23.7)	71 (43.6)	19 (27.9)	31 (28.7)	12 (21.4)	19 (36.5)	20 (27.4)	11 (31.4)
Separation anxiety	23 (8.6)	11 (8.1)	12 (9)	4 (10.5)	19 (11.7)	0 (0)	4 (3.7)	3 (5.4)	1 (1.9)	3 (4.1)	1 (2.9)
PTSD symptomatology	3 (1.1)	3 (2.2)	0 (0)	0 (0)	2 (1.2)	1 (1.5)	2 (1.9)	2 (3.6)	0 (0)	1 (1.4)	1 (2.9)
Sadness	42 (15.6)	22 (16.2)	20 (15)	4 (10.5)	29 (17.8)	9 (13.2)	24 (22.2)	12 (21.4)	12 (23.1)	16 (21.9)	8 (22.9)
Dysphoria	35 (13)	24 (17.6)	11 (8.3)	0 (0)	23 (14.1)	12 (17.6)	27 (25)	16 (28.6)	11 (21.2)	17 (23.3)	10 (28.6)
Negative self-esteem	10 (3.7)	7 (5.1)	3 (2.3)	0 (0)	8 (4.9)	2 (2.9)	9 (8.3)	4 (7.1)	5 (9.6)	5 (6.8)	4 (11.4)
Isolation	25 (9.3)	15 (11)	10 (7.5)	0 (0)	14 (8.6)	11 (16.2)	8 (7.4)	3 (5.4)	5 (9.6)	1 (1.4)	7 (20)
Nightmares	53 (19.7)	23 (16.9)	30 (22.6)	9 (23.7)	31 (19)	13 (19.1)	25 (23.1)	11 (19.6)	14 (26.9)	16 (21.99)	9 (25.7)
Headaches (headache)	17 (6.3)	11 (8.1)	6 (4.5)	1 (2.6)	12 (7.4)	4 (5.9)	16 (14.8)	8 (14.3)	8 (15.4)	8 (11)	8 (22.9)
Abdominal pain (stomach pain)	20 (7.4)	11 (8.1)	9 (6.8)	3 (7.9)	13 (8)	4 (5.9)	7 (6.5)	4 (7.1)	3 (5.8)	4 (5.5)	3 (8.6)
Skin problems	1 (0.4)	0 (0)	1 (0.8)	0 (0)	1 (0.6)	0 (0)	1 (0.9)	0 (0)	1 (1.9)	1 (1.4)	0 (0)
Dependence	42 (15.6)	25 (18.4)	17 (12.8)	6 (15.8)	34 (20.9)	2 (2.9)	7 (6.5)	3 (5.4)	4 (7.7)	4 (5.5)	3 (8.6)
Rejection by siblings and/or social	17 (6.3)	8 (5.9)	9 (6.8)	4 (10.5)	11 (6.7)	2 (2.9)	2 (1.9)	1 (1.8)	1 (1.9)	1 (1.4)	1 (2.9)
Jealousy	13 (4.8)	8 (5.9)	5 (3.8)	1 (2.6)	9 (5.5)	3 (4.4)	3 (2.8)	2 (3.6)	1 (1.9)	1 (1.4)	2 (5.7)
Obsessions	14 (5.2)	8 (5.9)	6 (4.5)	0 (0)	11 (6.7)	3 (4.4)	6 (5.6)	1 (1.8)	5 (9.6)	3 (4.1)	4 (8.6)
Strange behavior	4 (1.5)	3 (2.2)	1 (0.8)	0 (0)	4 (2.5)	0 (0)	0 (0)	0 (0)	0 (0)	0 (0)	0 (0)
Strange ideas	3 (1.1)	3 (2.2)	0 (0)	0 (0)	3 (1.8)	0 (0)	1 (0.9)	1 (1.8)	0 (0)	1 (1.4)	0 (0)
Attention problems	30 (11.2)	18 (13.2)	12 (9)	3 (7.9)	25 (15.3)	2 (2.99)	14 (13)	6 (10.7)	8 (15.4)	9 (12.3)	5 (14.3)
Restlessness	52 (19.3)	33 (24.3)	19 (14.3)	8 (21.1)	40 (24.5)	4 (5.9)	14 (13)	10 (17.9)	4 (7.7)	12 (16.4)	2 (5.7)
Impulsiveness	18 (6.7)	12 (8.8)	6 (4.5)	4 (10.5)	12 (7.4)	2 (2.9)	7 (6.5)	3 (5.4)	4 (7.7)	4 (5.5)	3 (8.6)
Disruptive behaviors	34 (12.6)	20 (14.7)	14 (10.5)	6 (15.8)	23 (14.1)	5 (7.4)	7 (6.5)	4 (7.1)	3 (5.8)	6 (8.2)	1 (2.9)
Aggressive behaviors	33 (12.3)	21 (15.4)	12 (9)	5 (13.2)	22 (13.5)	6 (8.8)	12 (11.1)	7 (12.5)	5 (9.6)	8 (11)	4 (11.4)
Low tolerance to frustration	48 (17.8)	32 (23.5)	16 (12)	8 (21.1)	34 (20.9)	6 (8.8)	18 (16.7)	10 (17.9)	8 (15.4)	11 (15.1)	7 (20)
Anger	22 (8.2)	16 (11.8)	6 (4.5)	3 (7.9)	16 (9.8)	3 (4.4)	12 (11.1)	7 (12.5)	5 (9.6)	9 (12.3)	3 (8.6)
Personal maladjustment	59 (21.9)	36 (26.5)	23 (17.3)	8 (21.1)	39 (23.9)	12 (17.6)	33 (30.6)	19 (33.9)	14 (26.9)	21 (28.8)	12 (34.3)
Maladjustment with mother	19 (7.1)	15 (11)	4 (3)	3 (7.9)	9 (5.5)	7 (10.3)	4 (3.7)	3 (5.4)	1 (1.9)	3 (4.1)	1 (2.9)
Maladjustment with father	28 (10.4)	11 (8.1)	17 (12.8)	4 (10.5)	16 (9.8)	8 (11)	9 (8.3)	8 (14.3)	1 (1.9)	6 (8.2)	3 (8.6)
Maladjustment with siblings	40 (14.9)	24 (17.6)	16 (12)	7 (18.4)	27 (16.6)	6 (8.8)	22 (20.4)	13 (23.2)	9 (17.3)	18 (24.7)	4 (11.4)
Sleeping problems	67 (24.9)	35 (25.7)	32 (24.1)	10 (26.3)	40 (24.5)	17 (25)	42 (38.9)	23 (41.1)	19 (36.5)	21 (28.8)	21 (60)
Feeding problems	16 (5.9)	9 (6.69)	7 (5.3)	2 (5.3)	11 (6.7)	3 (4.4)	8 (7.4)	2 (3.6)	6 (11.5)	2 (2.7)	6 (17.1)
Enuresis	4 (1.5)	4 (2.9)	0 (0)	0 (0)	4 (2.5)	0 (0)	2 (1.9)	2 (3.6)	0 (0)	2 (2.7)	0 (0)
Emotional instability	45 (16.7)	30 (22.1)	15 (11.3)	5 (13.2)	30 (18.4)	10 (14.7)	15 (13.9)	6 (10.7)	9 (17.3)	6 (8.2)	9 (25.7)
Learning difficulties	29 (10.8)	14 (10.3)	15 (11.3)	2 (5.3)	21 (12.9)	6 (8.8)	10 (9.3)	5 (8.9)	5 (9.6)	7 (9.6)	3 (8.6)
Others	14 (5.2)	6 (4.4)	8 (7.6)	0 (0)	7 (4.3)	5 (7.3)	3 (2.7)	0 (0)	3 (5.7)	0 (0)	4 (11.6)

When groups were compared by sex, statistically significant differences were observed regarding the perception of more psychological alterations by mothers regarding their sons and daughters, with higher rates perceived in males in the following variables: fear (χ21 = 5.223, *p* = 0.022, *V*_*Cramer*_ = 0.139); worry/nervousness (χ21 = 6.328, *p* = 0.012; *V*_*Cramer*_ = 0.153); dysphoria (χ21 = 5.223, *p* = 0.022; *V*_*Cramer*_ = 0.139); restlessness (χ21 = 4.294, *p* = 0.038; *V*_*Cramer*_ = 0.126); low frustration tolerance (χ21 = 5.987, *p* = 0.014; *V*_*Cramer*_ = 0.050); anger (χ21 = 4.711, *p* = 0.030; *V*_*Cramer*_ = 0.132); maladjustment with the mother (χ21 = 6.592, *p* = 0.010; *V*_*Cramer*_ = 0.157) and emotional instability (χ21 = 5.610, *p* = 0.018; *V*_*Cramer*_ = 0.144), although difference intensity among variables was low in all. No significant differences were found for the remaining variables (*p* > 0.05) (Table 4).

**TABLE 4:** Related variables with differences between sex according to mother’s perception in general emotional and/or behavioral effects.

	Chi-Square (V Cramer)
Fear	0.022 (0.139)
Worry/nervousness	0.012 (0.153)
Dysphoria	0.022 (0.139)
Restlessness	0.038 (0.126)
Low tolerance to frustration	0.014 (0.050)
Anger	0.030 (0.132)
Maladjustment with the mother	0.010 (0.157)
Emotional instability	0.018 (0.144)

In comparison of groups by age, rates of psychological alterations were generally higher in the youngest group (6 and 12 years). Based on information provided by mothers, statistically significant differences were obtained in the following variables: worry/nervousness (χ22 = 8.306, *p* = 0.016; *V*_*Cramer*_ = 0.176); restlessness (χ22 = 10.795, *p* = 0.005; *V*_*Cramer*_ = 0.200) and greater degree of dependence on the mother (χ22 = 11.693, *p* = 0.003; *V*_*Cramer*_ = 0.208), although difference in intensity was weak.

### Children’s Self-Perception of General Emotional and/or Behavioral Effects During Confinement

Global prevalence was calculated from the sum of scores on the Likert-type scale (1 = *somewhat*, 2 = *quite a lot*, 3 = *a lot*), as seen in Table 3. The one hundred and eight children who responded to the list of symptoms perceived that during lockdown they experienced an increase in 13 of the 35 variables on the checklist, specifically in sleep problems (38.9%); personal maladjustment (30.6%) and worry (28.7%); dysphoria (25%), nightmares (23.1%), sadness (22.2%), maladjustment in relationship with siblings (20.4%); worse tolerance to frustration (16.7%); headaches (14.8%); emotional instability (13.9%), attention problems and restlessness (13%); fear (12%) and aggressive behaviors and anger (11.1%).

When comparing groups by sex, boys reported greater maladjustment in their relationship with their father during confinement (Fisher’s exact test = 0.033; *V*_*Cramer*_ = 0.223), with low statistical significance difference. As for age, adolescents between 13 and 18 years old experienced more sleep problems (χ21 = 9.711, *p* = 0.002; *V*_*Cramer*_ = 0.300); eating (Fisher’s exact test = 0.014; *V*_*Cramer*_ = 0.257); and more emotional instability (χ21 = 6.054, *p* = 0.014; *V*_*Cramer*_ = 0.237), statistically significant with a weak difference intensity.

### Mothers’ Perception of Severe Emotional and/or Behavioral Effects of Confinement on Their Children

Severity of symptoms was assumed from the sum of scores on the Likert-type scale (2 = *quite a lot* and/or 3 = *a lot*). Results can be seen in Table 5. It should be noted that the percentage of severe symptoms perceived by the 185 mothers over the total number of children included in the sample during COVID-19 confinement was 3% or higher in various areas: Maladjustment with the mother and father, jealousy and abdominal pain (3%); anger (3.3%); maladjustment with siblings, isolation and sadness (4.1%); aggressive behaviors (4.5%); separation anxiety and attention problems (5.2%); fears, dysphoria and disruptive behaviors (5.6%); learning difficulties and personal maladjustment (5.9%); emotional instability, dependence and nightmares (6.7%); low tolerance to frustration (7.8%); sleep problems (10%); restlessness (11.5%); as well as worry/nervousness (16.7%).

**TABLE 5:** Perception of change in indicators of high psychological alteration during the period of confinement due to COVID-19.

Variables	Mothers’ perception (*N* = 269)						Children’ perception (*N* = 108)					
	f	%	Boys *n* = 136		Girls *n* = 133		f	%	Boys *n* = 56		Girls *n* = 52	
			f	%	f	%			f	%	f	%
Fear	15	5.6	10	7.4	5	3.8	5	4.6	3	5.4	2	3.8
Worry/nervousness	45	16.7	30	22.1	15	11.3	11	10.2	5	8.9	6	11.5
Separation anxiety	14	5.2	7	5.1	7	5.3	1	0.9	1	1.8	0	0
PTSD Symptomatology	1	0.4	1	0.7	0	0	1	0.9	0	0	0	0
Sadness	11	4.1	7	5.1	4	3	6	6.5	2	3.6	4	7.7
Dysphoria	15	5.6	12	8.8	3	2.3	7	6.8	5	8.9	2	3.8
Negative self-esteem	3	1.1	3	2.2	0	0	0	0	0	0	0	0
Isolation	11	4.1	8	5.9	3	2.3	1	0.9	1	1.8	0	0
Nightmares	18	6.7	10	7.4	8	6	9	8.3	4	7.1	5	9.6
Headaches	4	1.5	2	1.5	2	1.5	7	6.5	4	7.1	3	5.8
Stomachache	8	3	5	3.7	3	2.3	3	2.8	2	3.6	1	1.9
Skin problems	1	0.4	0	0	1	0.8	0	0	0	0	0	0
Dependence	18	6.7	10	7.4	8	6	2	1.9	1	1.8	1	1.9
Rejection by siblings and/or social	3	1.1	1	0.7	2	1.5	1	0.9	0	0	1	1.9
Jealousy	8	3	5	3.7	3	2.3	0	0	0	0	0	0
Obsessions	7	2.6	4	2.9	3	2.3	3	2.8	0	0	3	5.8
Strange behavior	2	0.7	2	1.5	0	0	0	0	0	0	0	0
Strange ideas	1	0.4	1	0.7	0	0	0	0	0	0	0	0
Attention problems	14	5.2	11	8.1	3	2.3	8	7.4	3	5.4	5	9.6
Anxiety	31	11.5	23	16.9	8	6	5	4.6	4	7.1	1	1.9
Impulsiveness	7	2.6	7	5.1	0	0	3	2.8	2	3.6	1	1.9
Disruptive behaviors	15	5.6	10	7.4	5	3.8	2	1.9	2	3.6	0	0
Aggressive behaviors	12	4.5	11	8.1	1	0.8	3	2.8	0	0	3	5.8
Low tolerance to frustration	21	7.8	16	11.8	5	3.8	3	2.8	2	3.6	1	1.9
Anger	9	3.3	8	5.9	1	0.8	3	2.8	1	1.8	2	3.8
Personal maladjustment	16	5.9	14	10.3	2	1.5	10	9.3	8	14.3	2	3.8
Maladjustment with mother	8	3	8	5.9	0	0	1	0.9	1	1.8	0	0
Maladjustment with father	8	3	3	2.2	5	3.8	2	1.9	2	3.6	0	0
Maladjustment with siblings	11	4.1	6	4.4	5	3.8	6	5.6	3	5.4	3	5.8
Sleeping problems	27	10	17	12.5	10	7.5	19	17.6	11	19.6	8	15.4
Feeding problems	6	2.2	2	1.5	4	3	0	0	0	0	0	0
Enuresis	2	0.7	2	1.5	0	0	1	0.9	1	1.8	0	0
Emotional instability	18	6.7	13	9.6	5	3.8	5	4.6	2	3.6	3	5.8
Learning difficulties	16	5.9	6	4.4	10	7.5	7	6.5	3	5.4	4	7.7
Others	5	1.9	3	2.1	2	0.16	2	1.8	0	0	2	3.8

When comparing groups by sex, statistically significant differences were observed regarding perception of alterations by mothers, higher prevalence being detected in males in the following variables: Worry/nervousness (χ21 = 5.610, *p* = 0.018; *V*_*Crame*_ = 0.144); dysphoria (χ21 = 5.509, *p* = 0.019; *V*_*Crame*_ = 0.103); attention problems (χ21 = 4.637, *p* = 0.03; *V*_*Crame*_ = 0.081); restlessness (χ21 = 7.831, *p* = 0.005; *V*_*Crame*_ = 0.124); impulsiveness (Fisher’s exact test = 0.014; *V*_*Crame*_ = 0.050); aggressive behaviors (χ21 = 8.492, *p* = 0.004; *V*_*Crame*_ = 0.75); low tolerance to frustration (χ21 = 5.987, *p* = 0.014; *V*_*Crame*_ = 0.050); anger (Fisher’s exact test = 0.036; *V*_*Crame*_ = 0.063); personal maladjustment (χ21 = 9.288, *p* = 0.002; *V*_*Crame*_ = 0.180); maladjustment with the mother (Fisher’s exact test = 0.007; V Cramer = 0.093), however, difference intensity between variables was generally weak. No significant differences were found for the remaining variables (*p* > 0.05) (Table 6). Likewise, differences linked to age could not be calculated, since the sample size was greatly reduced when disaggregating groups.

**TABLE 6:** Related variables with differences between sex according to mother’s perception in severe emotional and/or behavioral effects.

	Chi-Square/Fisher’s exact Test^1^ (V Cramer)
Worry/nervousness	0.018 (0.144)
Dysphoria	0.019 (0.103)
Attention problems	0.03 (0.081)
Restlessness	0.005 (0.124)
Impulsiveness^1^	0.014 (0.050)
Aggressive behaviors	0.004 (0.75)
Low tolerance to frustration	0.014 (0.050)
Anger^1^	0.036 (0.063)
Personal maladjustment	0.002 (0.180)
Maladjustment with mother^1^	0.007 (0.093)

*^1^Fisher’s exact test.*

Similarly, mothers did not report symptoms in 63.9% of children and adolescents, although they perceived that 17.1% presented between one and two indicators of serious alteration, and 19% three or more. From children who presented alterations, boys showed a greater number than girls with differences being statistically significant (*z* = −2.300, *p* = 0.021).

### Children’s Self-Perception of Severe Emotional and/or Behavioral Changes During Confinement

The rates of large changes reported by children and adolescents were 3% or higher in perception of fear, restlessness, and emotional instability (4.6%); maladjustment with siblings (5.6%); learning difficulties, headaches, and sadness (6.5%); dysphoria (6.8); attention problems (7.4%); nightmares (8.3%); personal maladjustment (9.3%); worry (10.2%); and sleep problems (17.6%). When comparing groups by sex, no statistically significant differences were observed, though there were some regarding age in the headache variable, more common in the group between 13 and 18 years old (Fisher’s exact test = 0.004; *V Cramer* = 0.300), confirming a difference of moderate intensity. No significant differences were identified for the remaining variables (*p* > 0.05) (Table 5).

Furthermore, children did not refer symptoms in 54.6% of cases. A percentage of 26.9% reported between one and two serious alterations, and 18.6% three or more indicators. No differences were found regarding sex or age (*p* > 0.05).

Finally, through Spearman’s Correlation Coefficient, a positive correlation was confirmed between the perception of the number of symptoms by mothers and children during confinement (*r_*s*_* = 0.593, *p* < 0.01).

## Discussion

This study assessed the perception of mothers on the general emotional and/or behavioral changes in their children during COVID-19 confinement. The perception of the minors themselves was also reported.

### Changes in Childhood Symptoms

The one hundred and eighty-five mothers who participated in the study reported an increase in previous problems in their children. The most common general effects were worry/nervousness (36.8%), sleep problems (24.9%), PTSD symptomatology and fear (24.5%). Likewise, a greater intensity in change was reported in some, 16.7% observed worry/nervousness, 11.5% restlessness, and 10% sleep problems.

Information provided by the children on the general emotional and/or behavioral effects of confinement revealed that from the 108, 38.9% had sleep problems and 30.6% personal maladjustment, these percentages were highest, followed by other symptoms such as worry/nervousness, dysphoria, nightmares, sadness, and maladjustment in relationships with siblings, as well as poorer tolerance to frustration, more headaches and emotional instability, attention problems and restlessness, fear, and aggressive behavior. From information provided by children on the serious emotional and/or behavioral effects of confinement, it must be noted that highest rates were observed in sleep problems (17.6%) and worry/nervousness (16.7%). Furthermore, only 18.6% of children reported three or more severe symptoms.

Finally, it is important to highlight the coincidence between the number of symptoms perceived by mothers and by children during confinement.

These variables were also reported in studies carried out on the general population, both in Spain (Berasategi et al., 2021; Amorós-Reche et al., 2022) and internationally (Pisano et al., 2020; Bignardi et al., 2021; Ravens-Sieberer et al., 2021; Weissman et al., 2021; Wright et al., 2021).

Nonetheless, it is difficult to compare percentages, since from the existing evidence in the community population it is unknown whether some of these children had any previous psychological problem or were at psychosocial risk. Furthermore, it should be added that various methodologies have been used in the studies reviewed, which aggravates the problem regarding comparison.

### Age and Sex Differences

As regards age and mothers’ perception of general effects, the group with the greatest impact was that of 6–12 years old, which differed significantly from other groups in symptoms related to worry/nervousness, restlessness, and dependence on the mother. As for perception of children and adolescents about general symptoms, it was observed that adolescents between 13 and 18 years old (older group) experienced more problems sleeping, eating and regulating emotions. Regarding severity of symptoms, statistically significant differences were seen in the headache variable in the group between 13 and 18 years old.

These results are consistent with contradictions reported to date by empirical evidence in the general population (Pearcey et al., 2020; Pisano et al., 2020; Lavigne-Cerván et al., 2021; Pizarro-Ruiz and Ordóñez-Camblor, 2021; Ravens-Sieberer et al., 2021), thus it is necessary to perform more exhaustive research that can provide information on the trajectories that these symptoms can follow when the pandemic disappears. The increase in symptomatology perceived by mothers in schoolchildren might be related to the greater time shared in the family context, in contrast to adolescents who usually take refuge in their room and in the use of social networks as a means of coping with isolation caused by confinement. In spite of this, adolescents perceive greater discomfort as regards psychosomatic symptoms.

As for differences by sex, mothers perceived that boys presented more symptoms of fear, dysphoria, restlessness, low tolerance to frustration, anger, maladjustment with the mother and emotional instability. Regarding symptoms, comparing groups by sex, statistically significant differences were observed in men, with the variables dysphoria, attention problems, restlessness, impulsiveness, aggressive behaviors, low tolerance to frustration, anger, and personal maladjustment and with the mother, with the largest differences founded. Moreover, boys exhibited a greater number of symptoms compared to girls.

When information was provided by the children themselves boys reported a higher degree of maladjustment in the relationship with the father during confinement However, in the area of serious affectation no differences were found regarding sex.

These results differ to those found in research of general population which highlight that girls generally present higher levels of anxiety, depression, and stress (Duan et al., 2020; Tamarit et al., 2020; Zhou et al., 2020; Carrillo-López et al., 2021; Gilsbach et al., 2021; Panchal et al., 2021). Therefore, it is necessary to delve further into what factors could influence these results in future research.

### Study Limitations

Some limitations in the present study should be taken into account. Firstly, the sample size is not excessively large and the sample has very specific characteristics, thus findings should not be generalized to other groups of children. Secondly, it is difficult to correctly estimate how pre-COVID-19 psychological problems have intensified, since comparable data is unavailable due to minors being in different treatment phases, and the fact that psychological evaluation was performed at the start. In addition, there are variables that have not been analyzed, such as the emotional state of mothers during the pandemic, which can influence their children’s perception of symptoms (Spinelli et al., 2020), as well as the reaction of children, quality of dyadic relationship prior to and during confinement, and the presence of resilience factors in mothers and children that help them cope with stressful situations.

## Conclusion

In summary, as other studies affirm (Odriozola-González et al., 2020; Ozamiz-Etxebarria et al., 2020; Wang et al., 2020), the health emergency caused by COVID-19 has had a serious impact on mental health in the adult population. It is a highly stressful vital event, due not only to confinement, unprecedented in Spanish society, but also as it was so unexpected. Evidence seems to confirm that effects are not insignificant in children and adolescents, with an even worse impact if adverse conditions prior to confinement are added.

In this study the importance is highlighted of information provided by children and adolescents on their psychological situation in a population exposed to IPV.

In this case, it is essential that professionals adapt both to circumstances arising from the pandemic and the specific characteristics of this population.

Previous studies (Jiao et al., 2020; Pisano et al., 2020; Zhou et al., 2020; Berasategi et al., 2021; Bignardi et al., 2021; Ravens-Sieberer et al., 2021; Weissman et al., 2021) have reported on the effects of confinement in the general population.

One of the main contributions of this study is that it is one of the first to provide information on the emotional and/or behavioral state of children and adolescents exposed to IPV during confinement, as well as differences in sex and age, through information from mothers and children. Its usefulness lies in the implications that can be derived regarding intervention in this population with such specific characteristics.

## Data Availability Statement

The raw data supporting the conclusions of this article will be made available by the authors, without undue reservation.

## Ethics Statement

Ethical review and approval was not required for the study on human participants in accordance with the local legislation and institutional requirements. Written informed consent to participate in this study was provided by the participants’ legal guardian/next of kin.

## Author Contributions

MA-L carried out the treatment protocol, collaborated on its application as clinical supervisor, corrected the evidence included in the research, and collaborated in the writing of the manuscript. MC collaborated performing statistical treatment, the writing of the manuscript, and the review of articles. AM-P, KN-M, and VF carried out part of the search for articles, writing of results, and preparation of tables and collaborated in the bibliographic search and writing of the introduction, results, and bibliographical references of the manuscript. CL-S designed the research and collaborated in the proofreading and writing of the manuscript. All authors contributed to the article and approved the submitted version.

## Conflict of Interest

The authors declare that the research was conducted in the absence of any commercial or financial relationships that could be construed as a potential conflict of interest.

## Publisher’s Note

All claims expressed in this article are solely those of the authors and do not necessarily represent those of their affiliated organizations, or those of the publisher, the editors and the reviewers. Any product that may be evaluated in this article, or claim that may be made by its manufacturer, is not guaranteed or endorsed by the publisher.
